# Consequences of Population Ageing on Health Systems: A Conceptual Framework for Policy and Practice

**DOI:** 10.4314/ejhs.v35i1.8

**Published:** 2025-01

**Authors:** Alireza Hajizadeh, Reza Hafezi, Fatemeh Torabi, Ali Akbari Sari, Maryam Tajvar

**Affiliations:** 1 Department of Health Management, Policy and Economics, School of Public Health, Tehran University of Medical Sciences, Tehran, Iran; 2 Department of Science and Technology Futures Studies, National Research Institute for Science Policy (NRISP), Tehran, Iran; 3 Department of Demography, Faculty of Social Sciences, University of Tehran, Tehran, Iran

**Keywords:** Conceptual Framework, Population Ageing, Health System, Functions, Goals

## Abstract

**Background:**

Population aging significantly affects the social, economic, and political landscapes of countries, including their health systems. This study aimed to develop a conceptual framework that illustrates the consequences of population aging on the functions and goals of health systems.

**Methods:**

This multi-method study consisted of four stages. First, the WHO-2000 framework for health systems was selected after a comprehensive review and consensus. Second, a systematic review identified the impacts of population aging. Third, an initial conceptual framework was designed. Finally, the framework was validated, completed, and finalized through semi-structured interviews.

**Results:**

The study identified 120 concepts related to the consequences of population aging, which were categorized within the functions and goals of the WHO framework. Key consequences for “stewardship” included adapting to demographic changes, modifying system design, and enhancing performance assessment. “Creating resources” faces increased demand, particularly for trained healthcare workers and geriatric care teams. “Financing” requires sustainable resources and strategic purchasing to address the higher healthcare costs associated with an aging population. “Service delivery” needs to focus on meeting the complex needs of older adults. The goals of health systems are also impacted, with implications for improving health outcomes, financial fairness, and responsiveness to non-medical expectations, including a client-oriented approach and respect for vulnerable older adults.

**Conclusion:**

Adopting strategies and policies based on these identified consequences, coupled with effective implementation, will help policymakers manage the impacts of population aging within health systems.

## Introduction

Population ageing is a consequence of “demographic transition,” defined by the United Nations Population Fund (UNFPA) as a country where more than 7% of the population is aged 60 and above (1). The global population aged 60 and over is expected to grow from approximately 900 million in 2015 (12% of the world's population) to over 1.2 billion by 2025 (2). By 2050, this population is projected to reach two billion, or 22% of the total population (3).

Population ageing significantly influences the social, economic, and political landscapes of nations and is often associated with rising disability and disease prevalence, which increases healthcare costs (4, 5). As these challenges become more widespread, ageing has emerged as a key concern, requiring effective policy, management, and planning interventions in health sectors (6, 7). Policymakers must develop strategies that provide social and economic support without viewing older individuals solely as a burden (8).

The impact of population ageing on health systems has been explored, particularly regarding rising healthcare costs linked to the increasing proportion of elderly individuals (9). For example, in the United States, people aged 65 and older accounted for 30% of healthcare spending in 2008, a figure expected to rise to 50% by 2030 (10). The global trend of population ageing has led social institutions to reassess service provisions, such as healthcare coverage, retirement plans, and social support systems (11-13).

While existing literature suggests that healthcare utilization patterns due to ageing are influenced by factors like lifestyle and chronic diseases (14), other consequences include changing family dynamics and increased use of care homes (15). These shifts can negatively affect health trends and health systems (16).

As physical and mental health tends to deteriorate with age, the demand for healthcare among the elderly increases, often requiring expensive medical technology and hospitalization (17). Specific frameworks have been proposed to help health systems adapt to population ageing, including the well-known “fit for ageing” approach (18), the responsiveness framework (19), the 4M framework for delivering high-quality care to older adults (20), and the integrated care for older people approach (21).

Despite extensive studies on the consequences of population ageing, few attempts have been made to consolidate and summarize these findings. This study aims to fill this gap by creating a conceptual framework that highlights the impacts of population ageing on health systems' functions and goals. We believe this framework will aid health managers and policymakers in addressing the challenges of an ageing population. The framework provides a structured approach for understanding, planning, and responding to demographic changes, ensuring that health systems are equipped to meet the diverse healthcare needs of older adults.

## Methods

**Figure 1:**
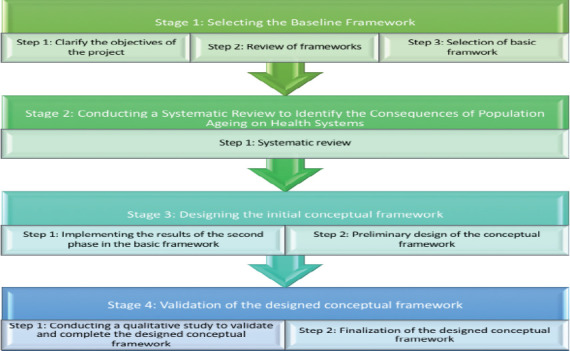
The four phases (8 steps) of the present multi-method study

**Study design**: This study employed a multi-method approach, consisting of four stages (Figure 1). Below is a detailed description of each stage.

**Stage 1: selecting the baseline framework:** An appropriate framework was needed to assess the impact of population ageing on health systems. In this stage, the research team reviewed internationally recognized frameworks for evaluating health system performance. Through consensus, the WHO 2000 health systems performance framework was chosen as the baseline framework for this study (22). This framework outlines three primary goals for health systems: improving health, ensuring responsiveness to nonmedical expectations, and promoting fairness in financial contributions. Achieving these goals relies on the effective execution of four core functions: stewardship, service delivery, resource creation, and financing (Including revenue collection, pooling, and purchasing).

**Stage 2: Systematic Review of Population Ageing Consequences:** A systematic review protocol was developed following the PRISMA-P guidelines (23) and registered with PROSPERO (CRD42022353165) on August 22, 2022. The review identified 23 relevant studies from an initial 1954 records. Full results of this review have been published elsewhere (24), but a summary is provided below:

**Eligibility criteria:**

Study types: Reviews, case studies, and original research were included
Date of publication: No time limitation
Language: Only English studies
Setting/Geography: No restrictions
Population: Studies focused on individuals aged 60 and above
Quality: Only high-quality studies were selected
Source: Peer-reviewed journals only.

Studies were excluded if they were unpublished, lacked primary data, or were short articles, editorial letters, or conference abstracts.

**Search strategy**: The team searched multiple databases, including PubMed, ProQuest, Web of Science, Scopus, and Google Scholar, using keywords related to population ageing. The research team also conducted reference snowballing to identify additional studies.

**Study selection and quality assessment:** Identified citations were imported into EndNote (version 8), and duplicates were removed. Titles, abstracts, and full texts were screened for inclusion. Two authors independently assessed study quality using the Joanna Briggs Institute (JBI) checklist (25), categorizing studies as low, moderate, or high quality.

**Data extraction and synthesis:** Information from included studies was extracted and analyzed through narrative synthesis. The data were mapped onto the WHO framework to determine how the findings impacted health system functions and goals.

**Stage 3: Designing the initial conceptual framework**

Based on the findings from the literature review and the WHO framework, the research team designed a conceptual framework that illustrates the consequences of population ageing on health systems. This conceptual framework serves as a roadmap for understanding the relationships between population ageing and health system components.

**Stage 4: Validation of the framework**

To validate the framework, the team conducted semi-structured interviews with 11 experts in population ageing and health systems. Feedback was analyzed and incorporated to refine the framework. Interviews continued until data saturation was reached, and a revised framework was sent for final approval. The validated framework incorporated expert feedback and was finalized.

## Results

**Figure 2:**
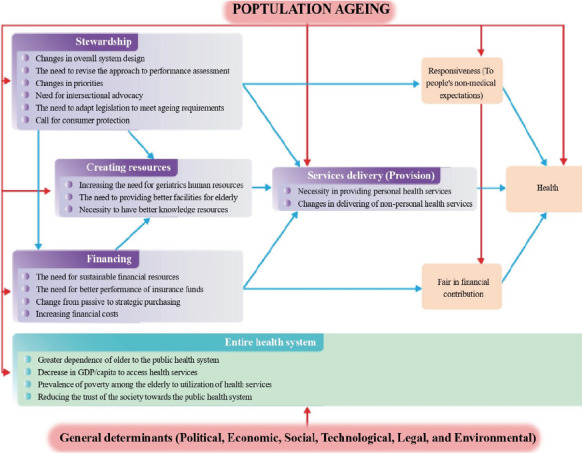
Conceptual framework for consequences of population ageing on health systems adopted from WHO 2000 framework

The study developed a conceptual framework that reveals the consequences of population ageing on health systems, building upon the WHO 2000 framework. The framework and its detailed components are shown in Figure 2 and Table 1, respectively. Below is a summary of the impacts on health system functions and goals:

**Table 1:** Consequences of Population Ageing on Functions and Goals of Health Systems

**Functions of Health Systems**	
Stewardship	**Changes in overall system design** The need for providing an interdisciplinary care model across specialties and health-care settings Necessity to emphasize on functional improvement The need to strengthen the role of the primary care system Raises doubts about the capability of the National Health System The need to increase consistency among related organizations Increasing private-public partnerships The need to strengthen the position of the Ministry of Health Growth of the private health sector **The need to revise the approach to performance assessment** Need to use the national accreditation system in elderly centers The need to evaluate cities based on components of age-friendly cities Call for evaluating health centres based on being Age-Friendly Health centres Increasing the need for sensitive indicators to monitor the health of the elderly The need to pay attention to the cost-benefit analysis of provider centers **Changes in priorities** The necessity for the integration of health and social care services The need to prioritize the elderly in the health system Attention to ageing as a criterion for prioritization **Need for intersectoral advocacy** Increasing the need for inter-sectoral cooperation The necessity for improving health insurance coverage and household income Need to use retirement plans Increasing demand from related sectors **The Need to adapt legislation to meet population ageing requirements** Urgent call for policy development The need for regulation in adjusting infrastructure The need to plan to address the challenges Recognition of older adults in the health policy framework The need for empowerment policies The need for policies to confront the induced demand of the private sector **Call for consumer protection** Higher need to provide social welfare More need to provide a safe environment with low levels of pollution Raise of physical inability and mobility limitation The need to activate non-governmental organizations related to ageing
Financing	**The need for sustainable financial resources** Facing a lack of resources to deal with ageing Need to launch the National Long-Term Care (LTC) Insurance Double whammy in public finance by ageing: increased health expenditures coupled with a reduction in tax revenue Reducing the financial independence of the elderly Need to provide adequate funding for healthcare delivery Reducing resources from insurance premiums **The need for better performance of insurance funds** Reducing the power of financial funds in pooling resources Reducing the fiscal balance of healthcare funds Much heavier financial burden for women than men **Change from passive to strategic purchasing** Influences on how to purchase health services Changing the package of health services Need for stakeholders' participation in the process of purchasing **Increasing financial costs** Increase in per capita costs for the age group Increase in the proportion of national expenditures Increase in out-of-pocket health expenditure Spending over one-third of the annual National Health Insurance (NHI) costs by elderly people Increase in health expenditures by the elderly for nursing services Higher cost in old patients than the middle age groups
Services Delivery	**Necessity in providing personal health services**: Influences were placed into two general categories: **Increasing demand for health services** Growth in specialty service demand Raising the prescription of diagnostic tests Increase in the number of outpatient and emergency department visits A rise in the percentage of inpatient days An increase in hospital surgeries An increase in hospice and palliative care services An increase the number of people receiving long-term care (LTC) An increasing demand for rehabilitation services An increasing demand for health consultations **Get complicated with service delivery** Increase in the number of elderlies with comorbidity Deterioration in mental and physical conditions Compatibility of quality of care with needs of older people The need for a holistic and integrated approach to providing services The need to integration of LTC services with health-care services The need to make more use of informal care Changes in the way health services are delivered Reducing the effectiveness of traditional service delivery models **Changes in the delivery of non-personal health services:** The general category was as follows: **Necessity of using national programs** Increasing the need for screening programs The need for mental and psychological protection programs in society The need to improve the lifestyles of older people Increasing the need for training to empower the elderly Increasing advertisements related to the awareness of the elderly on social media
Creating Resources	**Increasing the need for geriatrics human resources** High workload for health care workers More labor participation by women The need for the development of geriatrics The need for better training of health and social workers The need to create a more extensive integration between medical specialties Raise in demand for primary care physicians Additional demand for professional caregivers A much lower workforce participation rate among the elderly The increasing need for high performance geriatric care teams Reducing the active workforce in society The need to strengthen the communication skills of health workers **The need to provide better facilities for elderly (Physical resources such as facilities and equipment)** Require the reorganization and restructuring of hospital departments Necessitates to increasing healthcare facilities The need to establish Age-Friendly Centres Increasing inequity in the use of technology Reduced access of the elderly to assistive equipment Changes in the production and supply of drugs Increasing the average number of drug prescriptions Increases in consumption of medicine Growth in the price of medical equipment The need to establish more academic and research centers related to ageing Difficulty adapting the elderly to new technologies **Necessity to have better knowledge (Information) resources** Need for improvement in education systems Need for improvement in research systems Call for obtaining research grants for geriatrics The need for better data in ageing Increasing need for ageing databases
**Goals of health systems**	
Responsiveness	**The need to respect elderly people**: Respect for dignity Respect for confidentiality Respect for autonomy **Need to have a client orientation approach to**: Prompt attention Amenities of adequate quality Access to social support networks Choice of provider The need to increase the satisfaction of the elderly and their families Need to communicate verbally with the elderly Need for more responsibility in the health system Need to cover social services and professional care Increasing the phenomenon of loneliness among the elderly
Fairness in FC	Worsening of the out-of-pocket index Exposure of households to catastrophic healthcare expenditures Worsening in index of impoverishing health expenditure Increasing vulnerability of the elderly to economic shocks
Health	Raising the burden of diseases Higher prevalence of non-communicable diseases Increase in comorbidity Increasing inequality in the health status of the elderly Slowing down the speed of achieving universal health coverage (UHC) Increasing non-utilization of health services by the elderly Increasing gap between life expectancy index and healthy living index

### Consequences of population ageing on health system functions

**A) Stewardship:** The increasing proportion of individuals aged 60 and over brings demographic changes that impact stewardship at all levels of health system management. This includes adapting legislation, revising performance assessments, and advocating for intersectional approaches to meet the needs of older adults.

**B) Creating resources:** Population ageing drives the demand for additional healthcare workers, enhanced physical facilities, and increased research in geriatric care. Healthcare workers will face higher service demands, necessitating better training and the formation of specialized geriatric care teams. Additionally, healthcare facilities will need to adapt to accommodate an ageing population.

**C) Financing**: The ageing population increases healthcare costs, requiring sustainable financing strategies. Health systems must integrate resources, improve insurance fund performance, and shift to strategic purchasing to manage the increased financial burden effectively.

**D) Service delivery**: Service delivery must address the complex healthcare needs of older adults. This includes expanding both personal and non-personal health services tailored to the elderly.

### Consequences of population ageing on health system goals

**A) Improving health outcomes**: The ageing population challenges health systems to manage increased chronic conditions, comorbidities, and overall disease burden.

**B) Fairness in financial contributions**: Ageing exacerbates financial inequalities, increasing out-of-pocket costs, catastrophic expenditures, and impoverishing health costs.

**C) Responsiveness to non-medical expectations**: Health systems must become more client-oriented, ensuring that older adults are treated with respect and their non-medical needs are met. This includes responding to the vulnerabilities of older adults, particularly in terms of economic shocks.

## Discussion

Based on the WHO 2000 report, we propose a conceptual framework to illustrate the consequences of population ageing on the functions and goals of health systems. This framework aims to clarify the challenges posed by population ageing and highlight key considerations for improving health system management in response to this phenomenon.

As discussed in the results section, population ageing influences the stewardship function of health systems. These impacts necessitate changes in system structures, policymaking, prioritization, and the role of stakeholders involved in elderly care. For example, the increasing elderly population in Italy significantly impacted stewardship, highlighting the need for a redesign of the health system to prioritize public health initiatives (26). In Iran, key actions for better policymaking include revising existing structures, strengthening intersectoral cooperation, and implementing evidence-based policies (27). To address the needs of the ageing population effectively, interventions in health system functions are essential, with frameworks such as the elderly-friendly health system offering valuable guidance (28).

At the macro level, health policies should focus on minimizing healthcare expenditures for the elderly. Strategies such as training primary care doctors in geriatrics and promoting domiciliary care are vital (29). Population ageing impacts resource production, emphasizing the need for improved data, intersectoral collaboration, enhanced training for health and social workers, and the integration of older adults into health policymaking (30).

The financing function, as depicted in the framework, underscores the need for sustainable financial resources, improved performance of insurance funds, and strategic purchasing. Research conducted across 45 countries in 2021 showed that the rising health burden of the elderly increases healthcare expenditures and strains the fiscal balance of health funds (31). To cope with these challenges, a national long-term care (LTC) insurance plan is essential for ensuring seamless care and bridging the gap between healthcare and social welfare (32).

One of the main consequences of ageing, as shown in the framework, is the increasing financial burden. A 2017 study in Italy indicated that higher life expectancy and advanced age contribute to rising healthcare costs (26). Similar trends were observed in Taiwan, where individuals over 65 accounted for more than one-third of National Health Insurance (NHI) expenditures in 2010 (32).

The service delivery function also reflects the increasing demand for healthcare due to population ageing, which encompasses four dimensions: primordial and primary prevention, secondary prevention, tertiary prevention, and social support services (33). The complexity of service delivery increases with the elderly population, especially for those with comorbidities. As a result, there is a shift from disease-centered care to a more goal-oriented approach to ensure adequate healthcare (34). Additionally, the WHO emphasizes the need to reform LTC systems to address current and future needs of the elderly (35).

Population ageing also brings attention to the rising costs of medical care and LTC services. Changes in dependency ratios indicate an imbalance between the elderly and the working-age population, which will likely increase demand for social services and healthcare due to age-related conditions (36, 37).

Health system responsiveness to the needs of older adults is another important consideration. The adoption of an integrated responsiveness tool can enhance system efficiency and guide policy adaptations to accommodate ageing populations (38). Non-communicable diseases (NCDs) remain a key challenge, with an anticipated increase in the prevalence of cardiovascular diseases, cancer, diabetes, mental disorders, and chronic respiratory diseases (39, 40). The burden of NCDs and comorbidities is expected to rise, particularly in developing countries (41).

The goal of fair financial protection is also impacted by population ageing. A recent metaanalysis in China revealed that catastrophic health expenditure (CHE) increased from 13.0% in 2000 to 32.2% in 2020, with elderly individuals being a key driver (42). Households with elderly members are more vulnerable to CHE, resulting in greater financial strain compared to households with younger members (43).

Contextual factors such as political, cultural, economic, social, technological, legal, and environmental elements also play a significant role in how population ageing affects health systems. Health systems must adapt to rapid environmental changes to ensure sustainability (44).

In conclusion this study explores the implications of population ageing on health systems, building upon the WHO 2000 framework. The findings underscore the importance of recognizing the impact of population ageing on both the functions and goals of health systems. Health systems must provide comprehensive coverage and a wide range of services for the ageing population, addressing their non-medical needs and ensuring financial protection.

The developed conceptual framework serves as a tool for policymakers to better manage the impacts of population ageing within health systems. To track progress and effectively manage these impacts, the identification of relevant indicators is crucial. Future research should include comparative studies across different health systems, explore alternative frameworks such as the 2007 WHO health system building blocks framework (45), and analyze indicators related to fair financial protection as the population continues to age.

The following policy recommendations are proposed to address the consequences of population ageing on health systems. These strategies should be tailored to the specific needs and context of each health system:

**Long-term care planning**: Develop comprehensive long-term care strategies that integrate health and social services to support ageing in place.

**Healthcare workforce expansion:** Expand and train the healthcare workforce, including geriatric specialists, to meet the diverse needs of older adults.

**Integrated health and social services:** Foster collaboration between health and social service providers to offer holistic care.

**Financial sustainability**: Implement innovative financing mechanisms and cost-containment strategies to ensure financial sustainability and protect against catastrophic expenditures.

**Ageing-friendly health systems**: Design health systems that are accessible, respectful, and responsive to the needs of older adults.

**Chronic disease management**: Strengthen chronic disease management and prevention programs at the primary and secondary levels.

**Social safety nets**: Enhance social safety nets to shield elderly individuals from financial burdens.

**Community-Based Services**: Invest in community-based services and supports to enable ageing in place and promote well-being.

**Research and data:** Prioritize research on ageing-related health issues to inform evidence-based policymaking.

**Intergenerational Solidarity:** Promote intergenerational solidarity and respect, addressing ageism and discrimination.
